# Number of Physically Inactive Adults With Arthritis in the United States Who Could Improve Physical Function and Pain Control by Exercising

**DOI:** 10.5888/pcd17.200121

**Published:** 2020-09-03

**Authors:** George A. Kelley, Kristi S. Kelley, Leigh F. Callahan

**Affiliations:** 1 West Virginia University, Morgantown, West Virginia; 2 University of North Carolina, Chapel Hill, North Carolina

## Abstract

We estimated the number of physically inactive US adults with arthritis by state and nationally who could improve their physical function and pain control by participating in an exercise program. Our calculations were based on number-needed-to-treat, arthritis prevalence, physical inactivity, and 2010 US Census data. Estimates were lowest in the District of Columbia (physical function, n = 4,412; pain, n = 2,451) and highest in Texas (physical function, n = 325,504; pain, n = 180,835). Overall estimates were 4,119,792 for physical function and 2,288,771 for pain control. State-level estimates are important for allocating resources, public health program planning, and future research.

**Summary**

**What is already known?**

Exercise has been shown to improve physical function and pain control in adults with arthritis.

**What is added by this report?**

We provide state- and national-level estimates of the number of physically inactive adults with arthritis who can improve their physical function and pain control by exercising.

**What are the implications for public health practice?**

This information provides support for future studies on community-based exercise and arthritis, implementation of community-based exercise programs for adults with arthritis, and prioritization of resources aimed at such community-based exercise programs.

## Objective

Arthritis is a major public health problem in the United States, where it affects approximately 54.4 million adults (1). Physical function and pain control are 2 major problems in adults with arthritis (1,2). An estimated 23.7 million adults aged 18 or older with arthritis have arthritis-attributable activity limitations (1), and approximately 14.6 million report experiencing severe joint pain (3). In addition, more than half of adults with arthritis report persistent pain (4). Although exercise has been shown to improve physical function and reduce pain (5), physical activity levels in adults with arthritis are low, with state-level estimates of physical activity varying widely (2). Because national data may not be suitable for factors such as incidence, prevalence, and policy in each state, our objective was to provide overall and state-level estimates of the number of physically inactive adults with arthritis who could improve their physical function and pain control by participating in a community-based exercise program.

## Methods

We derived data for our study from 3 sources: 1) number-needed-to-treat (NNT) data from a previous meta-analysis that examined the effects of community-based exercise on physical function and pain in adults with arthritis aged 18 or older (5), 2) 2017 state-level estimates of the prevalence of arthritis and physical inactivity in adults with arthritis (2), and 3) state-level population data from the 2010 US Census (6). National estimates were also calculated.

We calculated the number of physically inactive adults with arthritis aged 18 or older who could improve their physical function and pain control by participating in a community-based exercise program by multiplying the reciprocal of the NNT by the number of physically inactive adults with arthritis in each state. The NNT represents the number of physically inactive adults with arthritis who would need to participate in a community-based exercise program for 1 person to improve physical function or pain control. Therefore, to calculate absolute estimates, we used the reciprocal of the NNT. For example, if the NNT for physical function is 5, then 1 in 5 (20%) physically inactive adults with arthritis would need to participate in a community-based exercise program for 1 person to benefit. For 10,000 physically inactive adults with arthritis, 2,000 (20% of 10,000) could improve their physical function by participating in a community-based exercise program. NNT, a well-established measure, presumes that not every individual participant in an intervention will experience its beneficial effects on a specific outcome. For example, a previous systematic review with meta-analysis reported that the NNT for adults with chronic musculoskeletal pain treated with topical nonsteroidal anti-inflammatory drugs (NSAIDs) was 5 for 1 (20%) to benefit (7). When using standardized mean difference effect sizes to calculate the NNT, the larger the effect size, the smaller the NNT and the potential for a greater number of participants to benefit (8). Details regarding the approach we used are available elsewhere (8).

We calculated NNT by using data from the meta-analysis of 33 randomized controlled trials representing 3,180 men and women (5) and a previously developed formula (8), with NNT values of 5 for physical function and 9 for pain derived from standardized mean difference effect size improvements of 0.34 for physical function and 0.20 for pain (5). Lower and upper 95% CIs were generated by following the same approach. State-level prevalence estimates for arthritis and physical inactivity, defined as a response of no to the question “During the past month, other than your regular job, did you participate in any physical activities or exercises such as running, calisthenics, golf, gardening, or walking for exercise,” were derived from 2017 Behavioral Risk Factor Surveillance System (BRFSS) data reported in a recent study (2) and state-level estimates for the number of adults aged 18 or older were obtained from 2010 US Census tables (6). These data were then used to calculate state-level prevalence estimates of arthritis and physical inactivity in adults with arthritis (2). Crude, rather than adjusted, estimates were used because age-standardized estimates are weighted to a standard population, and thus do not normally represent the percentage of individuals in the population when calculation of the absolute number of individuals is of interest. State-level data from the 2010 Census (6) were used because of the need to include the absolute number of all adults aged 18 or older in our calculations, data that were not available in the 2017 BRFSS report (2). For example, 2010 census data for the state of Alabama (6) with a focus on physical function showed 3,647,277 adults aged 18 or older, data that were not available in the 2017 BRFSS study (2). The population of 3,647,277 was then multiplied by the crude prevalence estimate of those with arthritis reported in the 2017 BRFSS data for Alabama (33.3%) (2). This resulted in an estimated 1,214,543 adults in Alabama with arthritis (3,647,277 × 0.333 = 1,214,543). We then multiplied the estimated number of adults in Alabama with arthritis (1,214,543) by the percentage of physically inactive adults in Alabama based on the 2017 BRFSS study (44.6%), arriving at an estimate of 541,686 (1,214,543 × 0.446). For physical function, the reciprocal of the NNT (1/5, or 0.20) from the authors’ prior meta-analysis (5) was multiplied by 541,686, to arrive at an average of 108,337 physically inactive adults with arthritis in Alabama who could improve their physical function by participating in a community-based exercise program. Unless noted, results are reported using absolute values. All data were analyzed by using Microsoft Excel 2016 (Microsoft Corp).

## Results

State-level estimates for the mean number of people who could improve their physical function by initiating a community-based exercise program ranged from a low of 4,412 (95% CI, 3,882–4,985) in the District of Columbia to a high of 325,504 (95% CI, 292,798–358,210) in Texas (Table). For pain control, estimates were also lowest in the District of Columbia (n = 2,451; 95% CI, 2,157–2,770) and highest in Texas (n = 180,835; 95% CI, 162,665–199,005). Across all states and the District of Columbia, the estimated mean number of physically inactive adults in the United States with arthritis who could improve their physical function by initiating a community-based exercise program was 4,119,792 (95% CI, 3,805,203– 4,444,217). For pain control, the estimate was 2,288,771 (95% CI, 2,114,000– 2,469,007).

**Table:** Estimated Number, by State, of Physically Inactive Adults With Arthritis Who Could Improve Their Physical Function and Pain Control by Exercising^a^

State	Physical Function, Mean (95% CI)	Pain Control, Mean (95% CI)
Alabama	108,337 (102,265–114,653)	60,187 (56,814–63,696)
Alaska	6,823 (5,694–8,070)	3,791 (3,163–4,483)
Arizona	79,861 (75,695–84,028)	44,367 (42,053–46,682)
Arkansas	55,311 (50,271–60,352)	30,728 (27,928–33,529)
California	290,046 (259,515–321,667)	161,136 (144,175–178,704)
Colorado	39,884 (36,466–43,466)	22,158 (20,259–24,148)
Connecticut	40,633 (37,831–43,563)	22,574 (21,017–24,202)
Delaware	13,484 (12,153–14,885)	7,491 (6,752–8,269)
District of Columbia	4,412 (3,882–4,985)	2,451 (2,157–2,770)
Florida	289,212 (269,393–309,031)	160,673 (149,663–171,684)
Georgia	140,253 (130,304–150,524)	77,919 (72,391–83,624)
Hawaii	13,356 (11,892–14,909)	7,420 (6,607–8,283)
Idaho	17,964 (16,145–19,837)	9,980 (8,970–11,021)
Illinois	144,988 (131,678–158,774)	80,549 (73,154–88,208)
Indiana	109,387 (104,402–114,648)	60,770 (58,001–63,694)
Iowa	37,299 (34,789–40,036)	20,721 (19,327–22,242)
Kansas	37,918 (36,279–39,456)	21,066 (20,155–21,920)
Kentucky	99,395 (93,183–105,607)	55,219 (51,768–58,671)
Louisiana	79,520 (73,389–85,652)	44,178 (40,772–47,584)
Maine	20,189 (18,747–21,696)	11,216 (10,415–12,054)
Maryland	77,491 (72,648–82,554)	43,051 (40,360–45,864)
Massachusetts	78,035 (69,284–87,273)	43,353 (38,491–48,485)
Michigan	161,890 (152,691–171,088)	89,939 (84,828–95,049)
Minnesota	52,054 (49,189–55,079)	28,919 (27,327–30,599)
Mississippi	55,861 (51,584–60,138)	31,034 (28,658–33,410)
Missouri	95,656 (89,059–102,253)	53,142 (49,477–56,807)
Montana	13,280 (12,108–14,530)	7,378 (6,727–8,072)
Nebraska	21,065 (19,752–22,443)	11,703 (10,973–12,468)
Nevada	28,016 (24,214–32,066)	15,564 (13,452–17,814)
New Hampshire	16,910 (15,383–18,601)	9,395 (8,546–10,334)
New Jersey	122,925 (114,298–131,551)	68,291 (63,499–73,084)
New Mexico	23,541 (21,358–25,801)	13,078 (11,866–14,334)
New York	236,780 (221,131–253,790)	131,545 (122,850–140,995)
North Carolina	127,436 (115,754–139,825)	70,797 (64,308–77,681)
North Dakota	8,891 (8,206–9,628)	4,940 (4,559–5,349)
Ohio	195,261 (184,498–206,535)	108,478 (102,499–114,742)
Oklahoma	64,951 (60,872–69,030)	36,084 (33,818–38,350)
Oregon	47,473 (43,057–52,047)	26,374 (23,921–28,915)
Pennsylvania	201,986 (185,781–218,770)	112,214 (103,212–121,539)
Rhode Island	15,938 (14,576–17,300)	8,855 (8,098–9,611)
South Carolina	71,663 (67,495–75,832)	39,813 (37,497–42,129)
South Dakota	8,958 (7,926–10,044)	4,977 (4,404–5,580)
Tennessee	118,250 (109,491–127,010)	65,695 (60,828–70,561)
Texas	325,504 (292,798–358,210)	180,835 (162,665–199,005)
Utah	20,166 (18,558–21,919)	11,203 (10,310–12,177)
Vermont	7,784 (7,097–8,527)	4,325 (3,943–4,737)
Virginia	112,946 (105,849–120,661)	62,748 (58,805–67,034)
Washington	63,215 (59,001–67,925)	35,119 (32,778–37,736)
West Virginia	47,569 (44,926–50,327)	26,427 (24,959–27,959)
Wisconsin	62,103 (55,425–69,003)	34,502 (30,792–38,335)
Wyoming	7,918 (7,222–8,614)	4,399 (4,012–4,786)
Overall	4,119,792 (3,805,203–4,444,217)	2,288,771 (2,114,000–2,469,007)

a Calculations based on 1) number-needed-to treat (NNT) data from a previous meta-analysis that examined the effects of community-based exercise on physical function and pain in adults ≥18 years of age with arthritis (NNT = 5 for physical function, NNT = 9 for pain control) (5), 2) 2017 state-level estimates of the prevalence of arthritis and physical inactivity in adults with arthritis (2), and 3) state-level population data from the 2010 US Census (6). For example, 2010 US Census data for the state of Alabama with a focus on physical function showed 3,647,277 adults aged ≥18, data that were not available in the 2017 Behavioral Risk Factor Surveillance System (BRFSS) study. The population of 3,647,277 was then multiplied by the crude prevalence estimate of those with arthritis reported in the 2017 BRFSS data for Alabama (33.3%). This resulted in an estimated 1,214,543 adults in Alabama with arthritis (3,647,277 × 0.333 = 1,214,543). We then multiplied the estimated number of adults in Alabama with arthritis (1,214,543) by the prevalence of physically inactive adults in Alabama based on the 2017 BRFSS study (44.6%), arriving at an estimate of 541,686 (1,214,543 × 0.446). The percentage to benefit, 20%, expressed as a proportion and derived as the reciprocal of an NNT of 5 from the authors’ prior meta-analysis, was multiplied by 541,686, to arrive at a mean of 108,337 physically inactive adults with arthritis in Alabama who could improve their physical function by participating in a community-based exercise program.

## Discussion

Our findings provide support for promoting community-based exercise programs to improve pain control and physical function in physically inactive adults with arthritis. These findings are similar to those previously reported for depression and anxiety (9). Although these findings should be helpful to researchers and practitioners, they may be especially useful for decision makers (eg, funding agencies, state legislators), because they provide state-level information for prioritizing resources aimed at community-based programs that may yield the greatest return on investment, several of which already exist for exercise (2). For example, at the national level, identification of the absolute number of physically inactive adults with arthritis in each state who may improve their physical function and pain control by participating in a community-based exercise program enables funding agencies to better allocate appropriate financial resources in support of such programs in each state. At the state level, decision makers such as state legislators have quantitative data (ie, number of people who could improve their pain control and physical function by participating in community-based exercise programs) that should be helpful when lobbying for resources to support programs designed to decrease the number of physically inactive adults with arthritis in their state.

Our findings should be interpreted along with relative state and national data regarding physically inactive adults with arthritis. Consideration of both absolute and relative data are important because the choice of one over the other may influence conclusions regarding the magnitude, direction, significance, and implications of the issue being addressed (10). Future research should consider generating similar absolute, state-level estimates based on other outcomes considered important in adults with arthritis, such as quality of life and fatigue (11,12). For example, Furner et al reported that overall, 27% of adults with arthritis reported fair or poor health compared with 12% of adults without arthritis (11). However, for those adults with arthritis who participated in recommended amounts of physical activity, prevalence was reduced to 19.2% (11). The prevalence of fatigue has also been shown to be greater among adults with arthritis than those without (12). Hootman et al reported that the number of adults who reported significantly greater fatigue on most days was higher among adults with arthritis (16.7%) than those without (7.2%) and among those reporting fatigue every day (12.2% vs 4.1%) (12). In addition, the prevalence of adults reporting fatigue every day was lower among those with arthritis who were physically active (12). Including estimates for such variables is considered important because it could help further demonstrate the multitude of outcomes that might be positively affected by engaging physically inactive adults with arthritis in a community-based exercise program rather than pharmacologic interventions such as NSAIDs. NSAIDs traditionally target one outcome (7), although that one outcome may be associated with improvements in other outcomes.

The major strength of our study is our use of different sources of information to arrive at state-level estimates of the number of physically inactive US adults with arthritis who could improve their physical function and pain control by exercising. However, our study has at least 5 potential limitations: 1) ecological fallacy, an inherent issue with aggregate data meta-analyses in which inferences regarding individual characteristics are made based on aggregate statistics, potentially making the inferences incorrect (13); 2) lack of state-level data to examine the potential impact of the uncertainty of NNT estimates that use sensitivity analyses, for example, use of the approach described by Furukawa (14) because of a lack of control group risk data for pain and physical function in community-based exercise studies; 3) limitations of BRFSS data (self-report bias, low response rates for some states, exclusion of institutionalized populations) (2); 4) lack of state-level data to examine subgroups (eg, sex, race/ethnicity, education, income); and 5) focus of the meta-analysis of randomized controlled intervention studies on structured community-based exercise (5), whereas data from the BRFSS were derived from the broader category of physical activity (2,15). With respect to the fifth limitation, the focus on community-based exercise programs in physically inactive adults with arthritis does not address the question of whether pain control and physical function could be improved if this population moved out of the category of being physical inactive, as defined by BRFSS (2), but did not meet the criteria for exercise (15). Thus, our findings may be underestimates given that our focus was on community-based exercise programs and not on the broader and more inclusive category of physical activity. Given these potential limitations, future research focused on state-level estimates of the benefits of exercise for improving physical function and pain control in adults with arthritis appears warranted, assuming necessary data are available.

In conclusion, our findings provide important state- and national-level information regarding the number of physically inactive adults with arthritis who could improve their physical function and pain control by participating in a community-based exercise program. This information should be helpful for allocating resources, public health program planning, and future research.
